# Germinal vesicle oocytes’ rescue: a worthless treatment or a patient-centred approach? Eight years of clinical experience in poor prognosis patients

**DOI:** 10.1093/humrep/deag115

**Published:** 2026-07-13

**Authors:** Danilo Cimadomo, Laura Escrich, Lucía Alegre, Noelia Grau, Arancha Galán, Cristina Rodríguez Varela, Marilena Taggi, Sara Pasku, Laura Rienzi, Ernesto Bosch, María-José Escribà

**Affiliations:** Laboratory of Biology and Biotechnology of Reproduction, Department of Biology and Biotechnology “Lazzaro Spallanzani”, University of Pavia, Pavia, Italy; IVIRMA Global Research Alliance, IVIRMA Italia, Rome, Italy; IVIRMA Global Research Alliance, IVI Valencia, Valencia, Spain; IVIRMA Global Research Alliance, IVI Valencia, Valencia, Spain; IVIRMA Global Research Alliance, IVI Valencia, Valencia, Spain; IVIRMA Global Research Alliance, IVI Valencia, Valencia, Spain; IVIRMA Global Research Alliance, IVI Foundation, Instituto de Investigaciones Sanitarias del Hospital La Fe de Valencia, Valencia, Spain; IVIRMA Global Research Alliance, IVI Foundation, Instituto de Investigaciones Sanitarias del Hospital La Fe de Valencia, Valencia, Spain; IVIRMA Global Research Alliance, IVIRMA Italia, Rome, Italy; Laboratory of Biology and Biotechnology of Reproduction, Department of Biology and Biotechnology “Lazzaro Spallanzani”, University of Pavia, Pavia, Italy; IVIRMA Global Research Alliance, IVIRMA Italia, Rome, Italy; Department of Biomolecular Sciences, University of Urbino “Carlo Bo”, Urbino, Italy; IVIRMA Global Research Alliance, IVI Valencia, Valencia, Spain; IVIRMA Global Research Alliance, IVI Foundation, Instituto de Investigaciones Sanitarias del Hospital La Fe de Valencia, Valencia, Spain; IVIRMA Global Research Alliance, IVI Valencia, Valencia, Spain; IVIRMA Global Research Alliance, IVI Foundation, Instituto de Investigaciones Sanitarias del Hospital La Fe de Valencia, Valencia, Spain

**Keywords:** GV rescue, GV oocyte, poor prognosis, low responder, rescue IVM

## Abstract

**STUDY QUESTION:**

Does germinal vesicle (GV) rescue by IVM provide a meaningful clinical benefit in patients with unexpectedly low mature oocyte yield, and which patient profiles are most likely to benefit?

**SUMMARY ANSWER:**

Especially in patients with more GV oocytes obtained, GV rescue increased the number of mature oocytes, zygotes, blastocysts, and euploid embryos per cycle, reduced transfer cancellation, and contributed to a 20% relative increase in cumulative live birth rate.

**WHAT IS KNOWN ALREADY:**

Immature oocytes retrieved during controlled ovarian stimulation are traditionally discarded due to their reduced developmental competence. Previous studies suggest that GV rescue may increase usable oocytes, but its clinical value, especially within a Pre-implantation Genetic Testing for Aneuploidies (PGT-A) framework, remains unclear.

**STUDY DESIGN, SIZE, DURATION:**

This retrospective clinical study analysed 8 years of GV rescue experience in a single centre (January 2017–December 2024), including 47 women (<39 years) undergoing ICSI with PGT-A who displayed suboptimal mature oocyte recovery (Metaphase II oocytes (MII) ≤6, maturity rate <80% relative to ≥14 mm follicles at ovulation trigger, and ≥4 GV oocytes). A sub-analysis of available time-lapse videos of rescued GV oocytes and sibling control MII oocytes was conducted to outline morphometric and morphodynamic determinants of rescue and blastulation.

**PARTICIPANTS/MATERIALS, SETTING, METHODS:**

All retrieved MII oocytes underwent ICSI, whereas GV oocytes were cultured in time-lapse systems for additional 24 h; those rescued (rMII) were inseminated. Outcomes included fertilization, blastulation, morphological quality, euploidy rates, and clinical outcomes after vitrified-warmed euploid blastocyst transfer. Patients’ benefit was defined as obtaining ≥1 euploid blastocyst from rMII. Morphometric features (relevant areas, diameters, perimeters) and morphodynamic parameters (timing of GV breakdown [GVBD], metaphase-I duration and first polar body (PB1) extrusion, second polar body (PB2) extrusion, pronuclei appearance, juxtaposition and fading, and cleavage) were annotated.

**MAIN RESULTS AND THE ROLE OF CHANCE:**

Among 266 GV oocytes, 53.7% matured to MII, increasing the available MII yield per patient by 75%. Fertilization rates were comparable between MII and rMII, while blastulation was significantly lower for rMII. Nevertheless, rMII-derived blastocysts showed similar morphology and euploidy to controls and contributed ≥1 additional euploid embryo in 11 patients. These patients exhibited higher total cumulus–oocyte complexes, GV count, and superior rMII fertilization and blastulation, although the demographics and basal parameters as well as the sibling control MII competence were comparable. GV rescue contributed to an 8% relative increase in the number of patients with euploid blastocysts and to a 20% relative increase in the number of patients with a live birth. Successfully rescued GV oocytes were substantially larger and underwent faster GVBD. rMII oocytes were significantly smaller than sibling control MII oocytes. Among the former, earlier pronuclear juxtaposition and shorter cleavage timings were associated with successful blastocyst development.

**LIMITATIONS, REASONS FOR CAUTION:**

The design is retrospective at a private IVF centre. The sample size is small, although this is the consequence of purposely strict inclusion criteria. Findings apply to patients with unexpectedly low MII yield undergoing PGT-A cycles in experienced laboratories.

**WIDER IMPLICATIONS OF THE FINDINGS:**

GV rescue represents a patient-centred strategy that can increase cumulative IVF success in cycles with limited mature oocyte yield. Further refining of patient selection criteria and standardizing this approach may enhance its clinical utility in future appraisals. The definition of artificial intelligence-powered pipelines to extract relevant morphometric/morphodynamic features to inform future predictive tools along with improvements in rescue protocols represent important upcoming innovations in this area of research.

**STUDY FUNDING:**

The study was partially supported by the ESHRE Research Grant 2024.

**DISCLOSURES:**

The authors declare no conflict of interest related with the content of this manuscript.

**TRIAL REGISTRATION NUMBER:**

Not applicable.

## Introduction

Ovarian stimulation aims to optimize ovarian response and, therefore, the number of mature oocytes available per aspirated follicle. In comparison to a natural cycle, the stronger ovarian response obtained after treatment with gonadotrophins increases the chances of achieving a healthy live birth (Sunkara *et al.*, 2011; Drakopoulos *et al.*, 2016).

For ART purposes, only metaphase II (MII) oocytes are used, while immature forms, namely metaphase I (MI) and germinal vesicle (GV) oocytes (with a prevalence of ≈5% and ≈10%, respectively) (Taggi *et al.*, 2026), are usually discarded from an ongoing IVF cycle because of their low developmental competence (Bartolacci *et al.*, 2024). The proportion of mature oocytes expected to be retrieved per total number of follicles measured on the day of ovulation trigger depends on follicular size (Abbara *et al.*, 2018); for example, when the follicle diameter ≥14 mm, 70–80% oocytes are expected to be retrieved at the MII stage (ESHRE and Alpha, 2017). In general, 95% of women retrieve nearly 80% oocytes which are at the MII stage after ovarian stimulation. Conversely, 5% of IVF patients might retrieve a number of MII oocytes that does not match what is expected from the follicle count at ovulation trigger, either because of ovulation or due maturity rate lower than expected (Ben-Shlomo *et al.*, 1991; Işik and Vicdan, 2000; Zreik *et al.*, 2000; Aktas *et al.*, 2005; Castillo *et al.*, 2020; Taggi *et al.*, 2026). These scenarios clearly compromise the expected success rate per cycle. To counteract this, the number of MII oocytes available could be increased by rescuing immature oocytes, as suggested by several authors (for a review, see Bartolacci *et al.*, 2024).

It is well known that up to 60% of the GV oocytes retrieved after ovarian stimulation may progress to the MII stage in different culture media and within different timeframes (Soler *et al.*, 2025). Rescued MII oocytes can then be fertilized by ICSI and progress to the blastocyst stage, eventually giving rise to healthy live births after transfer (Nagy *et al.*, 1996; Lee *et al.*, 2016; Escrich *et al.*, 2018). Still, rescue *in vitro* maturation (rescue IVM) is considered controversial because of the lower clinical performance of rescued oocytes compared to their sibling control MII, and its routine application is therefore discouraged in the general IVF population (Coticchio *et al.*, 2025). Yet, the recent update of the Istanbul Consensus also states ‘immature oocytes could be considered in case of poor prognosis individuals/couples and/or when alternatives are not available’ (Coticchio *et al.*, 2025). This caution, which has been expressed also across other documents published by international and national scientific societies (e.g. ESHRE (Lundin *et al.*, 2023) and SIFES-MR (Società Italiana di Fertilità e Sterilità e Medicina della Riproduzione) (Cimadomo *et al.*, 2025)) reflects the uncertainty regarding which patients may benefit from rescue IVM, whose practice may involve additional clinical workload and costs.

The present study aimed at retrospectively outlining the efficacy of GV rescue strategy across an 8-year timeframe (2017–2024) in terms of relative contribution to IVF success in a selected population of poor prognosis patients. A comprehensive description of all embryological and clinical outcomes is also provided to compare the competence of r-MII oocytes versus their sibling control MII.

## Materials and methods

This is a retrospective study of 8 years of clinical experience (from January 2017 to December 2024) with GV rescue in a Pre-implantation Genetic Testing for Aneuploidies (PGT-A) program at a private IVF centre in Spain (IVIRMA Global Research Alliance, IVI Valencia). The project was approved by the ethics committee of the Instituto Universitario IVI (Valencia, Spain; reference number: 2510-VLC-128-ME).

**Figure 1.**
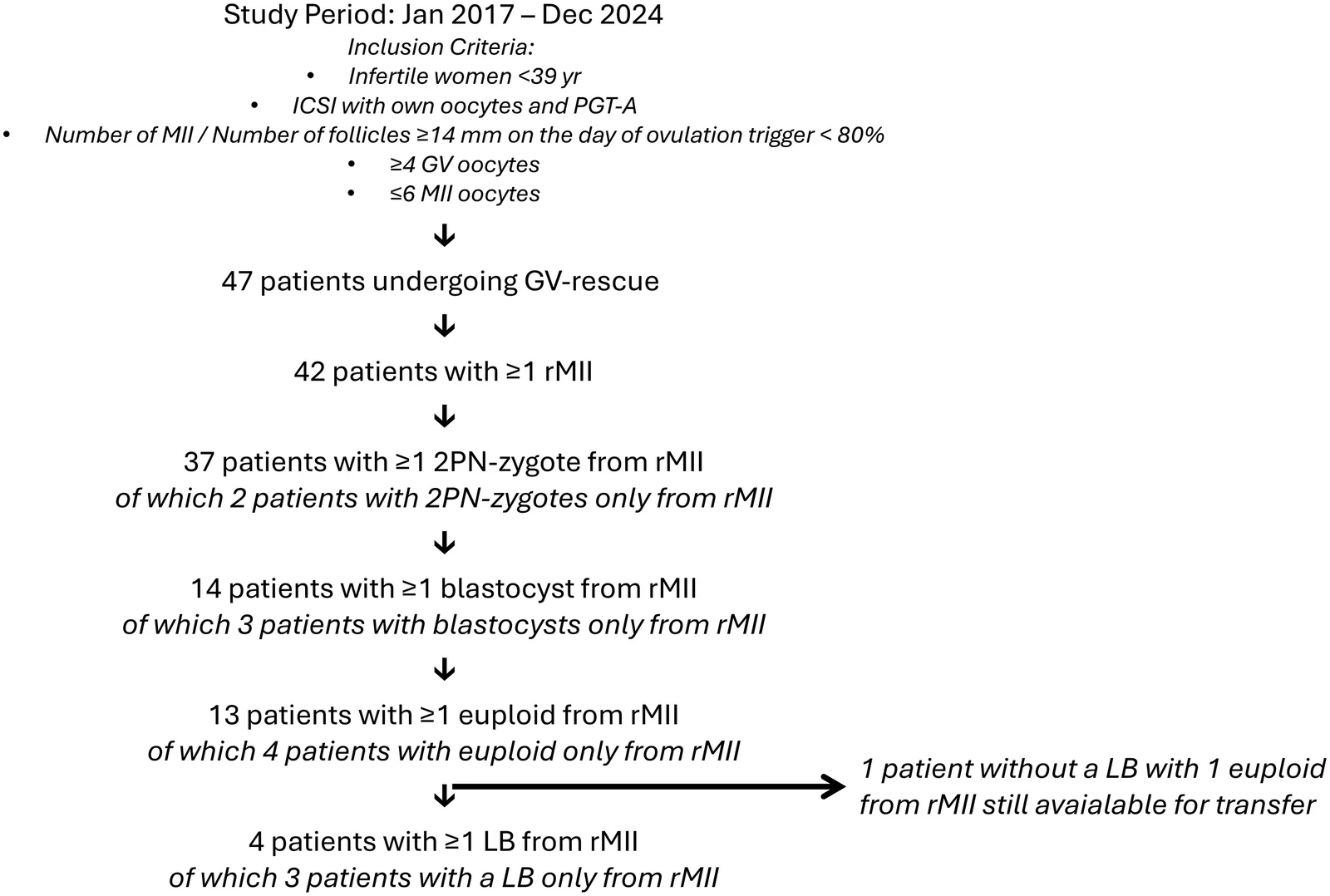
**Study flowchart**. MII, metaphase II; PGT-A, preimplantation genetic testing for aneuploidies; GV, germinal vesicle; PN, pronuclei; LB, live birth.

The study included infertile women younger than 39 years planned for an ICSI cycle with PGT-A, showing a yield of MII oocytes lower than 80% of the number of follicles ≥14 mm counted at the time of ovulation trigger, who collected less than six MII oocytes and at least four GV oocytes. Basal anti-Müllerian hormone (AMH) levels and antral follicle count (AFC) were measured on cycle days 1–3, while oestradiol, progesterone, and follicles ≥14 mm were recorded on the day of ovulation trigger. Embryological and clinical outcomes were recorded in a relational database. FORT (Follicular Output Rate, calculated as number of pre-ovulatory follicles/number of antral follicles at baseline ×100) and FOI (Follicle-to-Oocyte Index, calculated as the number of retrieved oocytes per number of antral follicles at baseline ×100) were also noted. Figure 1 shows the study flowchart.

### Ovarian stimulation, oocyte retrieval, and ICSI

All patients included in the study underwent ovarian stimulation using a daily protocol of GnRH antagonist for pituitary down-regulation and recombinant FSH alone or combined with recombinant LH or hMG. No specific inclusion criteria were defined based on the ovarian stimulation protocol, and the choice of a protocol was made on a case-by-case basis, according to patients’ characteristics and clinician preference. Concerning ovulation induction, it was performed with a standard dosage of hCG, with GnRH-agonist or with a combined administration of triptorelin and hCG. Thirty-six hours after induction, oocytes were retrieved by follicular aspiration. Cumulus–oocyte complexes (COCs) were cultured for 4 h, after which surrounding cumulus cells were removed. Oocytes were then assessed for maturity, those at the MII stage were selected for ICSI, whereas immature oocytes at the GV stage were selected for rescue. MI oocytes were discarded.

### GV rescue approach

For GV rescue, we followed the protocol described in Escrich *et al.* (2018) and recently improved by Soler *et al.* (2025). In brief, cumulus-free GV oocytes were cultured in time-lapse slides (EmbryoScope, Vitrolife, Sweden or Geri, Genea-Biomedx, Australia) containing 25 µl of CSCM-NXC per drop (FUJIFILM Irvine Scientific, USA) overlaid with 1.2 ml of mineral oil (Sidney IVF, CooperSurgical, USA). Embryo culture was performed in EmbryoScope (Vitrolife, Sweden) or Geri (Genea-Biomedx, Australia) incubators and lasted a maximum of 24 h at 37 °C, 5% CO_2_ and 5% O_2_. GV oocytes that had progressed to the MII stage within the first 24 h of culture (rescued MII, rMII) were selected for subsequent fertilization by ICSI within 4 h from the first polar body extrusion; otherwise, oocytes that had not reached the MII stage were discarded (Escrich *et al.*, 2012, 2018). For ICSI purposes, the same sperm as the day before was used after being kept at room temperature and briefly incubated for 1 h.

### Embryo culture

Microinjected oocytes of both origins (retrieved mature or rescued) were individually cultured for 5 or 6 days in continuous Gems culture medium (Genea-Biomedx, Australia). After 16–20 h, oocytes were assessed for fertilization. On Day 5 or 6, blastocysts derived from oocytes of both origins were graded according to ASEBIR criteria (Cuevas Saiz *et al.*, 2018) and biopsied for subsequent PGT-A analysis at an external laboratory. Biopsied blastocysts were individually vitrified (Cobo *et al.*, 2012).

### Endometrial preparation for cryopreserved embryo transfer

All patients displayed ovarian function and underwent down-regulation for pituitary suppression with a GnRH agonist (Decapeptyl 3.75 mg i.m., single dose, Ipsen, France). Treatment began on Day 2/3 of menstruation with estradiol valerate administered either orally (Progynova, Bayer, Germany; 6 mg/day) or transdermal, with patches (Evopad, Janssen Cilag, Belgium; 150 µg every 2 days). After 10–14 days on estrogens, a vaginal ultrasound was performed to measure endometrial thickness and to confirm a triple layer pattern (Labarta *et al.*, 2017).

Patients were considered ready for transfer when endometrial thickness was >6.5 mm, ovaries were quiescent (detected by ultrasound), serum estradiol (E2) was >100 ng/ml, and serum progesterone level was <1 pg/ml. Vaginal micronized exogenous progesterone (Utrogestan, SEID, Italy; Progeffik, Effik, Switzerland) was administered 5 days before transfer, at a dose of 400 mg twice a day. The last dose before blastocyst transfer (10th dose) was administered on the morning of the day of transfer, ∼6 h beforehand. In the case of pregnancy, hormonal replacement treatment was maintained until week 12.

Only single euploid vitrified-warmed blastocyst transfers were performed. Priority was given to euploid blastocysts derived from sibling control MII oocytes, whereas rMII-derived blastocysts were transferred only when no sibling transferable embryos were available.

### Outcome measures

All embryological outcomes (fertilization rate, blastulation rate, euploidy rate, and euploid blastocyst rate per inseminated oocyte [main embryological outcome]) were reported per patient for sibling control MII oocytes and for rMII oocytes, separately. The relative contribution of GV-rescue to the number of MII oocytes, zygotes with two pronuclei (2PN), blastocysts, and euploid blastocysts was reported among patients with ≥1 sibling control MII oocyte, ≥1 2PN-zygote from sibling control MII, ≥1 blastocyst from sibling control MII, ≥1 euploid blastocyst from sibling control MII. Data were shown also for patients obtaining only 2PN-zygotes, blastocysts, and euploid blastocysts from rMII oocytes. Data were compared on a per oocyte or embryo basis, on a per patient basis overall, and on a per patient basis through a paired approach whenever each developmental milestone was reached through both sibling control MII and rMII oocytes. Inner cell mass (ICM) and trophectoderm (TE) morphological quality and day of development were also compared in blastocysts from sibling control MII and rMII. Clinical outcomes were also reported by comparing sibling control MII and rMII oocytes. Specifically, positive pregnancy test rate was calculated as the percentage of patients having a quantitative serum value of ß-hCG ≥50 IU/l on Day 11 after transfer. Biochemical pregnancy loss rate was defined as positive pregnancy tests that did not result in the visualization of a gestational sac with foetal heartbeat at the scan. Miscarriage rate was defined as a pregnancy loss within 22 gestational weeks per clinical pregnancy. Live birth rate was defined as an ongoing pregnancy beyond 22 gestational weeks per transfer. At last, cumulative live birth was defined as patients with ≥1 live birth among concluded cycles (i.e. live birth achieved or no live birth achieved and no embryo available for transfer) (Zegers-Hochschild *et al.*, 2017a,b). Gestational age and birthweight were also reported among newborns. Finally, to explore the relative contribution of GV rescue to all clinical outcomes, we reported the patients with ≥1 inseminated oocyte, ≥1 2PN zygotes, ≥1 blastocyst, ≥1 euploid blastocyst, ≥1 live birth (main clinical outcome), >1 live birth, ≥1 live birth and additional embryos available, no live birth and additional embryo available without and with the contribution of rMII.

### Definition of patients who benefit from the GV rescue approach

To ascertain under which clinical and demographic scenarios women would or would not obtain some advantage from the GV rescue approach, patients were retrospectively grouped based on whether they obtained ≥1 euploid blastocyst from rMII or not. The former were considered as having had benefit from the procedure.

### Morphometric and morphodynamic GV rescue analyses

**Figure 2.**
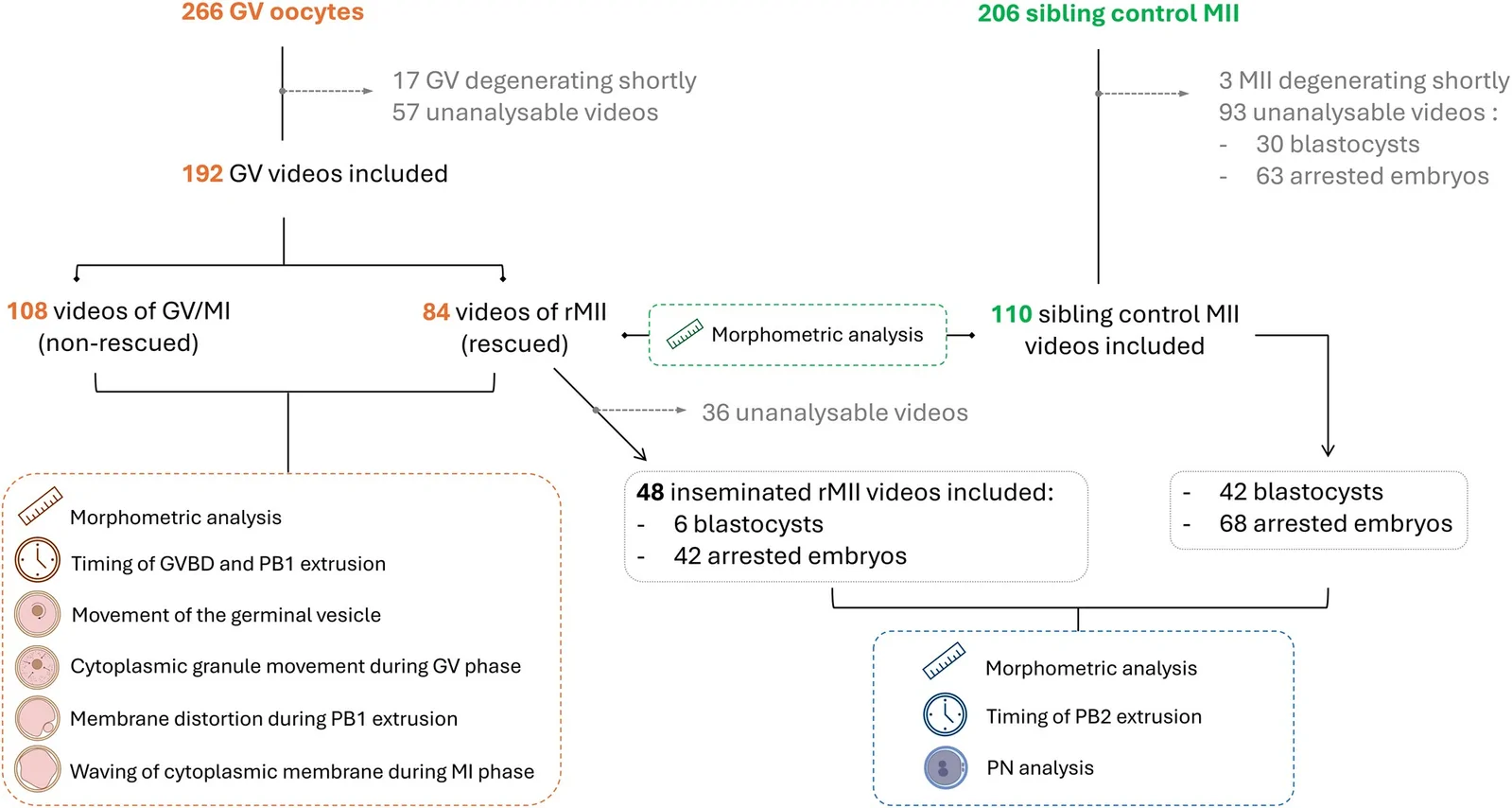
**Flowchart summarizing the videos included in the morphometric and morphodynamic analyses**. GV, germinal vesicle; MI, metaphase I; MII, metaphase II; rMII, MII derived from GV rescue; GVBD, germinal vesicle breakdown; PB1, first polar body; PB2, second polar body; PN, pronuclei.

Morphometric and morphodynamic assessments were conducted on a selected subset of available time-lapse recordings extracted from EmbryoScope incubators to evaluate their association with maturation and blastulation outcomes after GV rescue. Some videos were excluded because unanalysable (e.g. presence of bubbles in the well, time gaps in the acquisition). The overall workflow, including the selection process, the number of recordings included in each analysis, and the morphometric and morphodynamic variables investigated, is summarized in Fig. 2. Morphometric measurements were manually performed using ImageJ software at the GV or MII stage. The area, perimeter, and diameter of the oocyte including the zona pellucida (ZP) and of the oocyte proper, excluding the ZP, were quantified, along with ZP thickness. In addition, the area, perimeter, and diameter of the GV and of the first polar body (PB1) were measured at the GV and MII stages, respectively. Morphodynamic parameters included the timing of GV breakdown (GVBD), metaphase-I duration, and PB1 extrusion, defined as the time elapsed from the beginning of observation to each respective event. The behaviour of the GV prior to GVBD was classified as stable or moving, according to the presence of detectable intracellular movement, and its position at breakdown was categorized as central or cortical based on its localization within oocyte cytoplasm (Supplementary Fig. S1A). Cytoplasmic granule dynamics during the GV stage were qualitatively evaluated according to the presence of coordinated granule movement (Supplementary Fig. S1B). Finally, membrane-related features were assessed, including membrane waving during the MI stage (Supplementary Fig. S1C), defined as oscillatory oolemma movements occurring prior to completion of meiosis I, and membrane distortion during PB1 extrusion, defined as transient oolemma deformation associated with PB emission (Supplementary Fig. S1D). The following fertilization dynamics were annotated: second polar body (PB2) extrusion, pronuclear (PN) appearance, PN juxtaposition, PN fading, and time to first cleavage. PB2 extrusion was defined as the time from insemination to extrusion of the second polar body. PN appearance corresponded to the first visualization of two distinct pronuclei, whereas PN juxtaposition was defined as the alignment and close apposition of the pronuclei before syngamy. PN fading was recorded as the disappearance of PN, indicating syngamy completion. Time to cleavage was defined as the interval from ICSI to the first mitotic division resulting in the 2-cell embryo stage. Further annotations beyond fertilization were not conducted due to limited sample size in the rMII group.

### Statistical analysis

Categorical variables were expressed as number of cases (n/N) and rate (%), and comparisons were carried out according to oocyte origin (sibling control MII or rMII) using the Fisher’s exact test. Continuous variables were first assessed for their normal distribution using the Shapiro–Wilk test and then reported as either mean ± SD or as median with first (Q1) and third (Q3) quartiles. Statistically significant differences were determined using *t*-tests or Mann–Whitney *U*-tests, as appropriate.

Intracohort comparisons in patients obtaining both rMII and sibling control MII were conducted through paired *t*-tests. Regression analyses were performed to assess the association between selected morphometric and morphodynamic features and oocyte maturation outcomes following GV rescue and to define the variables associated with the chance of obtaining ≥1 euploid blastocyst after GV rescue. A *P*-value equal to or lower than 0.05 was considered statistically significant. The software SPSS v19 (USA) was used for statistics.

## Results

### Embryological and clinical outcomes

**Figure 3.**
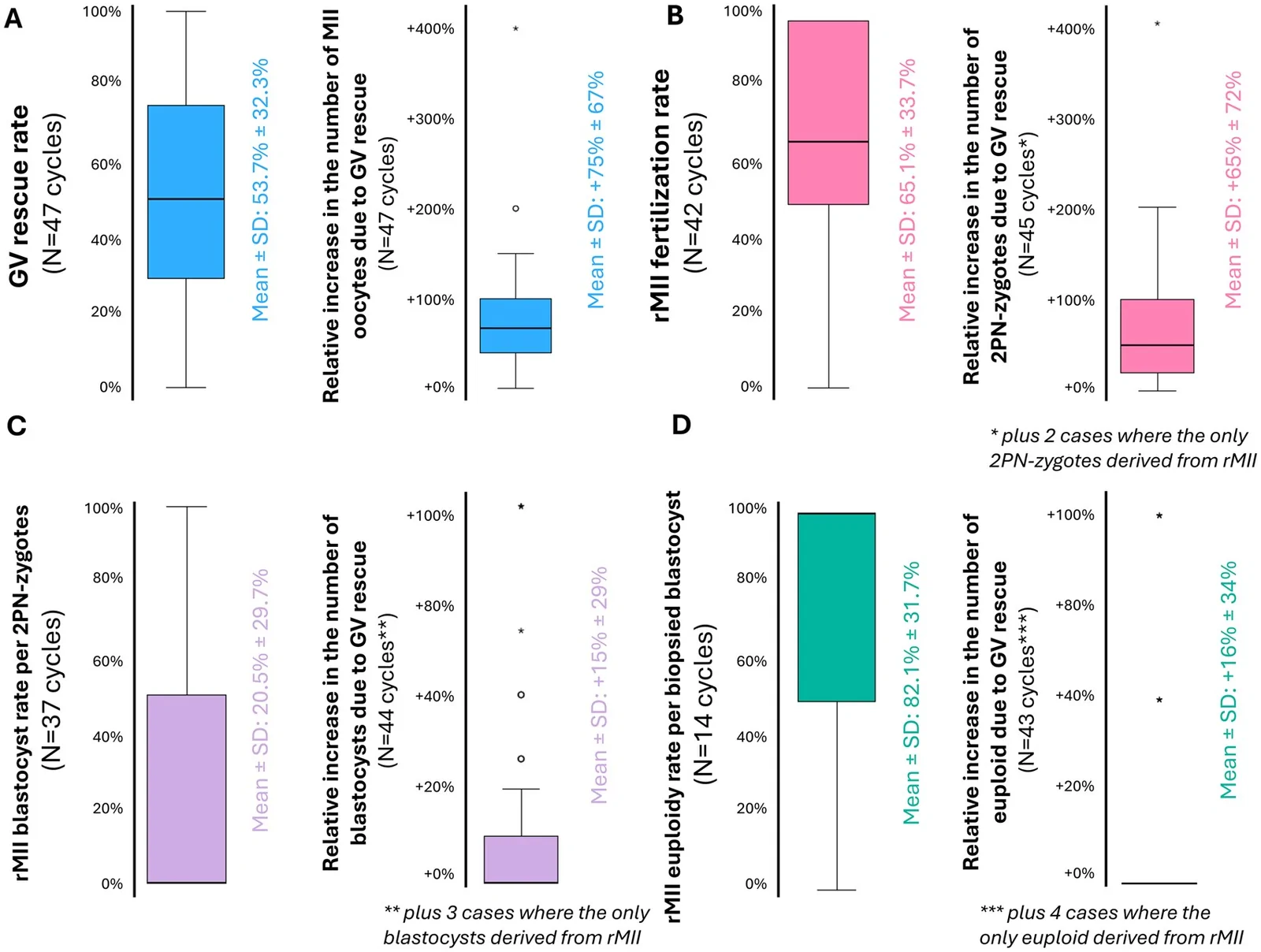
**Outcomes of germinal vesicle (GV) rescued oocytes (rescued metaphase II, rMII) and their relative contribution per cycle**. Box plots and relative mean ± SD illustrate the performance of rMII across consecutive developmental milestones from maturation to euploid blastocyst formation. Left graphs show each outcome per cycle, while right graphs show the relative increase in MII oocytes, 2PN zygotes, blastocysts, and euploid blastocysts due to GV rescue in cycles with ≥1 corresponding control MII-derived outcome. **(A)** GV rescue rate was conducted in 47 cycles; **(B)** r-MII oocytes were inseminated in 42 cycles and contributed to the only 2PN zygotes obtained in 2 cycles; **(C)** 2PN zygotes derived from rMII were cultured in 37 cycles and contributed to the only blastocysts obtained in 3 cycles; **(D)** Blastocysts derived from rMII were biopsied in 14 cycles and contributed to the only euploid blastocysts obtained in 4 cases.

GV rescue was conducted over an 8-year period in 47 patients who met the established strict inclusion criteria. This cohort represents 1.7% of all women undergoing ICSI for PGT-A during the study period (47 of 2769 patients). Their detailed characteristics and the overall embryological outcomes are presented in Table 1. Out of a total of 266 GV oocytes (5.7 ± 2.4 per patient that derived from a total GV rate of 51.5% and an average GV rate per patient of 52.4%±12.9%), 127 progressed to rMII within 24 h of culture (2.7 ± 1.5 per patient resulting in a total rescue rate of 47.7% and a mean rescue rate per patient of 53.7%±32.3%). Rescued-MII oocytes increased the number of MII oocytes available for ICSI per patient from 4.4 ± 1.6 to 7.1 ± 2.4, representing an average 75 ± 67% increase (Fig. 3A).

**Table 1.** Demographics and embryological outcomes of the 47 patients who underwent germinal vesicle (GV) rescue in the study period.

Age (years) mean ± SD	N = 47 patients, 35.8 ± 2.4
AMH (ng/ml) mean ± SD	N = 47 patients, 2.2 ± 1.7
AFC	
N	538
Mean ± SD	N = 47 patients, 11.5 ± 4.7
Main cause of infertility, n/N, %	
Idiopathic	8/47, 17%
AMA	32/47, 68.1%
Endometriosis	2/47, 4.3%
Adenomyosis	1/47, 2.1%
Tubal	1/47, 2.1%
Reduced ovarian reserve	3/47, 6.4%
Severe male factor, n/N, %	
No	37/47, 78.7%
Yes	10/47, 21.3%
Ovulation trigger, n/N, %	
hCG	12/47, 25.5%
GnRH-a	23/47, 48.9%
Double trigger	12/47, 25.5%
Follicles ≥14 mm at ovulation trigger	
N	424
Mean per patient ± SD	N = 47 patients, 9.0 ± 3.7
FORT (Follicles ≥14 mm at ovulation trigger/AFC)	
Mean per patient ± SD	N = 47 patients, 83.5% ± 23.2%
COCs	
N	516
Mean per patient ± SD	N = 47 patients, 11.0 ± 3.8
FOI (COCs/AFC) mean per patient ± SD	N = 47 patients, 126.5% ± 30.0%
Sibling control MII retrieved	
N	206
Mean per patient ± SD	N = 47 patients, 4.4 ± 1.6
Recovery rate (MII/follicles ≥14 mm at ovulation trigger)	
Overall	206/424, 48.6%
Mean per patient ± SD	N = 47 patients, 49.4% ± 17.5%
GV retrieved	
N	266
Mean per patient ± SD	N = 47 patients, 5.7 ± 2.4
GV rate (GV/COCs)	
Overall	266/516, 51.5%
Mean per patient ± SD	N = 47 patients, 52.4% ± 12.9%
rMII	
N	127
Mean per patient ± SD	N = 47 patients, 2.7 ± 1.5
Rescue rate (rMII/GV)	
Overall	127/266, 47.7%
Mean per patient ± SD	N = 47 patients, 53.7% ± 32.3%
Total MII (Sibling control MII + rMII)	
N	333
Mean per patient ± SD	N = 47 patients, 7.1 ± 2.4
2PN from sibling control MII	
N	147
Mean per patient ± SD	N = 47 patients, 3.1 ± 1.5
2PN from rMII	
N	84
Mean per patient ± SD	N = 47 patients, 1.8 ± 1.3
Total 2PN	
N	231
Mean per patient ± SD	N = 47 patients, 4.9 ± 2.0
Sibling control MII fertilization rate	
Overall	147/206, 71.4%
Mean per patient ± SD	N = 46 patients, 73.6% ± 25.6%
**Table 1. Continued**	
rMII fertilization rate	
Overall	84/127, 66.1%
Mean per patient ± SD	N = 42 patients, 65.1% ± 33.7%
Blastocysts from sibling control MII	
N	72
Mean per patient ± SD	N = 47 patients, 1.5 ± 1.2
Blastocysts from rMII	
N	17
Mean per patient ± SD	N = 47 patients, 0.4 ± 0.6
Total blastocysts	
N	89
Mean per patient ± SD	N = 47 patients, 1.9 ± 1.5
Sibling control MII blastulation rate	
Overall	72/147, 49%
Mean per patient ± SD	N = 44 patients, 47.1% ± 31.8%
rMII blastulation rate	
Overall	17/84, 20.2%
Mean per patient ± SD	N = 37 patients, 20.5% ± 29.7%
Euploid from sibling control MII	
N	37
Mean per patient ± SD	N = 47 patients, 0.8 ± 0.8
Euploid from rMII	
N	13
Mean per patient ± SD	N = 47 patients, 0.3 ± 0.5
Total euploid	
N	50
Mean per patient ± SD	N = 47 patients, 1.1 ± 1.0
Sibling control MII euploidy rate	
Overall	37/72, 51.4%
Mean per patient ± SD	N = 36 patients, 53.7% ± 42.3%
rMII euploidy rate	
Overall	13/17, 76.5%
Mean per patient ± SD	N = 14 patients, 82.1% ± 31.6%
Euploid blastocyst rate per sibling control MII	
Overall	37/206, 18%
Mean per patient ± SD	N = 46 patients, 18.8% ± 21.6%
Euploid blastocyst rate per rMII	
Overall	13/127, 10.2%
Mean per patient ± SD	N = 42 patients, 12.4% ± 24%
Euploid blastocyst rate per GV	
Overall	13/266, 4.9%
Mean per patient ± SD	N = 47 patients, 4.5% ± 8.0%

AMH, anti-Müllerian hormone; AFC, antral follicle count; COC, cumulus-oocyte-complexes retrieved; FORT, follicular output rate; FOI, follicle-to-oocyte index; MII, metaphase-II oocytes; rMII, MII derived from GV rescue; 2PN, two pronuclei.

**Figure 4.**
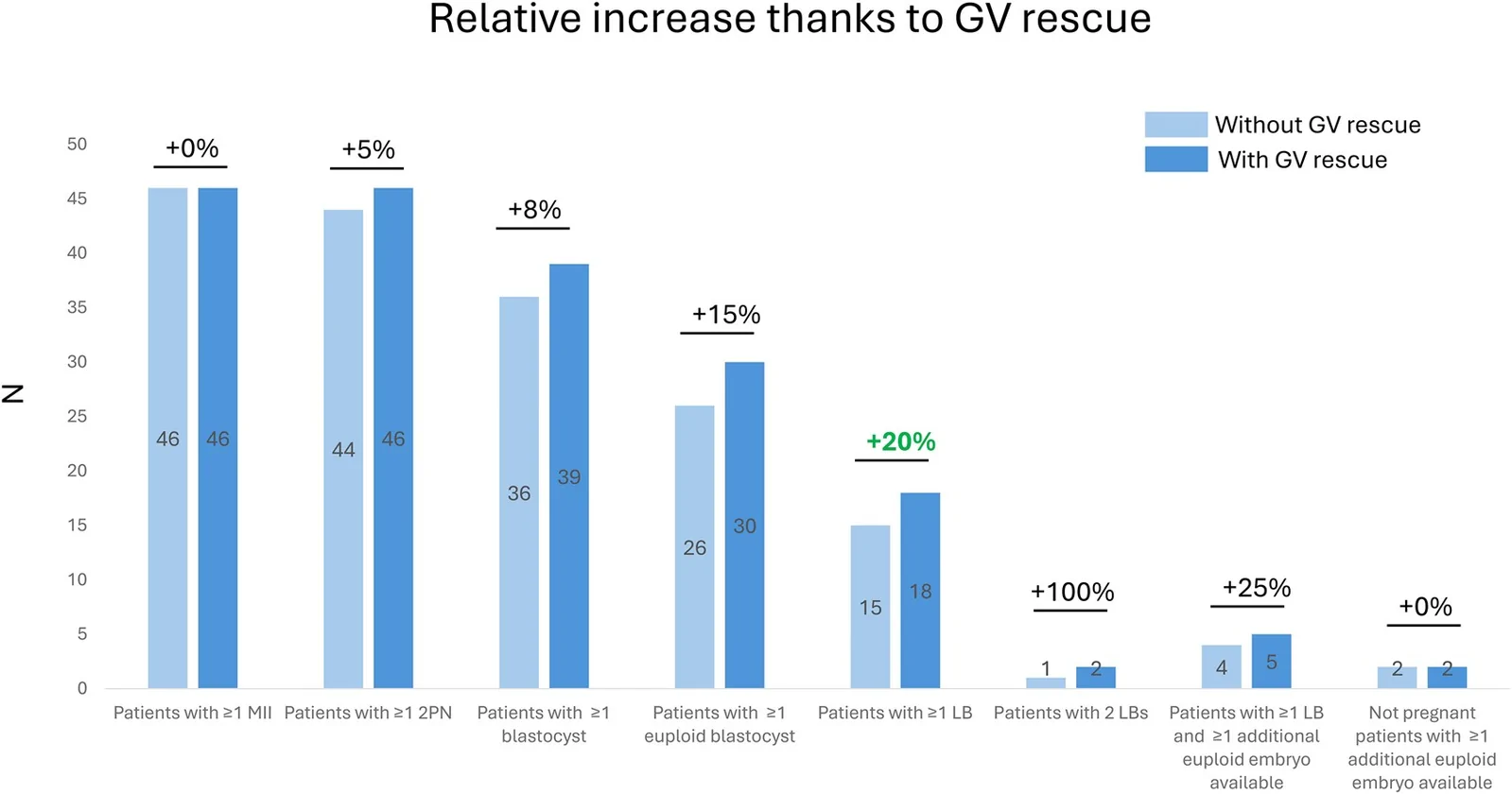
**Relative contribution of the germinal vesicle oocyte rescue strategy (GV rescue) to all cycle outcomes**. All percentages represent the relative increase in the number of patients who showed a benefit from GV rescue at each stage across the IVF cycle. The light blue bars represent the number of patients without considering GV rescue, whereas the dark blue bars show the corresponding number of patients after the application of GV rescue. The percentages above each pair of bars indicate the relative increase attributable to GV rescue. The green text indicates the primary clinical outcome, namely the increase in the number of patients achieving at least one live birth following GV rescue. MII, metaphase II; PN, pronuclei; LB, live birth.

Fertilization rates were comparable between sibling control MII (N = 206) and rMII (N = 127) oocytes, with no difference from an overall analysis, from a per patient analysis (N = 46 vs N = 42 patients) and from a paired analysis (N = 42 patients) (Table 2). The overall rMII fertilization rate was 66.1% with a mean per patient of 65.1 ± 33.7% resulting into a 65 ± 72% average relative increase in the number of 2PN zygotes obtained thanks to GV-rescue in 45 cycles (from 3.1 ± 1.5 to 4.9 ± 2.0) plus 2 cycles where the only 2PN zygotes derived from GV-rescue (Fig. 3B; Tables 1 and 2). The relative contribution of GV rescue to obtaining at least one 2PN-zygote determined an increase from 44 to 46 patients (+5%) (Fig. 4).

**Table 2.** Main embryological and clinical outcomes in control metaphase II (MII) compared to rescue-MII (rMII) oocytes.

	Control MII oocytes	rMII oocytes	Statistics
LABORATORY OUTCOMES			
Fertilization rate			
*Overall 2PN/inseminated, %*	147/206, 71.4%	84/127, 66.1%	*P* = 0.330
*Mean per patient ± SD*	N = 46, 73.6% ± 25.6%	N = 42, 65.1% ± 33.7%	*P* = 0.322
*Mean 2PN per patient with both MII and rMII ± SD*	N = 42, 73.4% ± 25.4%	N = 42, 65.1% ± 33.7%	Delta: +8.3%, 95% CI −6.1% to +22.7%, *P* = 0.252
Blastulation rate			
*Overall blastocysts/2PN, %*	72/147, 49.0%	17/84, 20.2%	*P* < 0.001
*Mean per patient*	N = 44, 47.1% ± 31.8%	N = 37, 20.5% ± 29.7%	*P* < 0.001
*Mean per patient with 2PNs from both MII and rMII ± SD*	N = 35, 44.3% ± 31.7%	N = 35, 18.3% ± 29.0%	Delta: +26%, 95% CI +12.5% to +39.5%, *P* < 0.001
Overall ICM morphological quality, n/N*, %*			
A	7/72, 9.7%	0/17, 0%	
B	45/72, 62.5%	14/17, 82.4%	*P* = 0.221
C	20/72, 27.8%	3/17, 17.6%	
Overall TE morphological quality, n/N*, %*			
A	1/72, 1.4%	1/17, 5.9%	
B	37/72, 51.4%	11/17, 64.7%	*P* = 0.261
C	34/72, 47.2%	5/17, 29.4%	
Overall day of blastocyst development, n/N*, %*			
5	50/72, 69.4%	12/17, 70.6%	*P* = 0.999
6	22/72, 30.6%	5/17, 29.4%	
Euploidy rate per biopsied			
*Overall euploid/biopsied, %*	37/72, 51.4%	13/17, 76.5%	*P* = 0.101
*Mean per patient ± SD*	N = 36, 53.7% ± 42.4%	N = 14, 82.1% ± 31.7%	*P* = 0.040
*Mean per patient with blastocysts from both MII and rMII ± SD*	N = 11, 58.3% ± 36.8%	N = 11, 81.8% ± 33.7%	Delta: −23.5%, 95% CI −57.1% to +10%, *P* = 0.149
Euploid blastocyst rate per inseminated			
*Overall euploid blastocysts/inseminated, %*	37/206, 18.0%	13/127, 10.2%	*P* = 0.101
*Mean per patient ± SD*	N = 46, 18.8% ± 21.6%	N = 42, 12.4% ± 24.0%	*P* = 0.064
*Mean per patient with both MII and rMII ± SD*	N = 42, 19.8% ± 21.9%	N = 42, 12.4% ± 24.0%	Delta: +7.4%, 95% CI −3.3% to +18.1%, *P* = 0.171
Euploid blastocyst rate per GV			
*Overall euploid blastocysts/GV, %*	–	13/266, 4.9%	–
*Mean per patient ± SD*		N = 47, 4.5% ± 8.0%	
Overall PGT-A result, n/N*, %*			
*Euploid*	37/72, 51.4%	13/17, 76.5%	
*Single aneuploidy*	21/72, 29.2%	3/17, 17.6%	*P* = 0.269
Complex aneuploidy	9/72, 12.5%	1/17, 5.9%	
Partial aneuploidy	5/72, 6.9%	0/17, 0%	
CLINICAL OUTCOMES			
Overall positive pregnancy test rate per transfer n/N*, %*	20/31, 64.5%	6/10, 60%	*P* = 0.999
Overall biochemical pregnancy rate per positive pregnancy test n/N*, %*	1/20, 5%	2/6, 33.3%	*P* = 0.123
Overall miscarriage rate per clinical pregnancy n/N*, %*	3/19, 15.8%	0/4, 0%	*P* = 0.999
Overall live birth rate per transfer n/N*, %*	16/31, 51.6%	4/10, 40%	*P* = 0.719
Overall gestational age *Mean ± SD*	39.3 *±* *1.3 weeks*	40.3 *±* *0.5 weeks*	*P* = 0.122
Overall birthweight *Mean ± SD*	3463 *±* *421 g*	3440 *±* *107 g*	*P* = 0.750

The comparisons are conducted per oocyte or embryo (Fisher’s or chi-squared tests), per patient (*t*-tests or Mann–Whitney *U*-tests) and per patient with oocytes and embryos at each developmental stage obtained from both control MII and sibling rMII (paired *t*-tests).

2PN, two pronuclei; ICM, inner cell mass; TE, trophectoderm; GV, germinal vesicle; PGT-A, preimplantation genetic testing for aneuploidies.

Blastulation rates were significantly lower for rMII than for sibling control MII oocytes from an embryo-based perspective (20.2% vs 49%) (Table 2), a patient-based perspective (N = 37, 20.5 ± 29.7% vs N = 44, 47.1 ± 31.8%), and a paired analysis among patients with both 2PN from MII and from rMII (N = 35, 18.3 ± 29.0% vs 44.3 ± 31.7%) (Tables 1 and 2). The embryos derived from GV-rescue involved a 15 ± 29% average relative increase in the number of blastocysts obtained, specifically from 1.5 ± 1.2 using only MII oocytes to 1.9 ± 1.5 (Fig. 3C). Interestingly, in three cycles, the only blastocysts obtained derived from rMII. The relative contribution of GV-rescue to obtaining at least one blastocyst involved an increase from 36 to 39 patients (+8%) (Fig. 4). Importantly, the morphological quality of both ICM and TE, as well as the day of biopsy, were comparable between sibling control MII- and rMII-derived blastocysts (Table 2).

Overall, 13 euploid blastocysts were obtained starting from 266 GV oocytes, resulting in a 4.9% rate and corresponding to a 4.5%±8.0% average rate per patient (Tables 1 and 2). Euploidy rate per biopsied blastocyst did not show significant differences between embryos derived from rMII and sibling control MII oocytes (76.5% vs 51.4%) (Tables 1 and 2). Likewise, the distribution according to the specific PGT-A diagnoses was comparable (Table 2). On a per patient basis, a mildly significantly higher euploidy rate was reported among rMII-derived blastocysts than among sibling control MII-derived ones (N = 14, 82.1 ± 31.7% vs N = 36, 53.7 ± 42.4%, *P* = 0.04), but this difference was not statistically significant from a paired analysis among the 11 patients obtaining blastocysts from both rMII and MII (Tables 1 and 2). When the euploid blastocyst rate was reported per inseminated oocyte, no significant difference was shown from all analyses, including the euploid blastocyst rate per sibling control MII versus paired cohorts of r-MII (N = 42 patients, 19.8 ± 21.9% vs 12.4 ± 24.0%, *P* = 0.171) (Tables 1 and 2). Overall, 37 euploid blastocysts were obtained from sibling control MII oocytes with a mean 0.8 ± 0.8 per patient (Table 1). This number increased to 50 (overall mean 1.1 ± 1.0 per patient) thanks to GV rescue, which corresponds to an average 16 ± 34% relative increase in 43 patients. In four cycles, the only euploid blastocysts were obtained from rMII oocytes (Fig. 3D). The relative contribution of GV-rescue to obtaining at least one euploid blastocyst involved an increase from 26 to 30 patients (+15%) (Fig. 4).

Table 2 shows the clinical outcomes after single vitrified-warmed euploid blastocyst transfers derived from sibling control MII versus rMII. All results were comparable, including the gestational age and birthweight of the babies born. The relative contribution of GV rescue to the cumulative live birth was +20% as the patients delivering at least one live birth increased from 15 to 18 (Fig. 4).

To outline the factors associated with a clinical benefit derived from GV rescue, patient characteristics and embryological outcomes were compared between those who did not obtain euploid blastocysts from rMII (n = 34) versus those who did (n = 13). No significant difference was observed in age, AMH, AFC, main causes of infertility, prevalence of severe male factor, or type of ovulation trigger (Table 3); conversely, patients obtaining euploid blastocysts from rMII oocytes retrieved significantly more COCs (13.5 ± 5.1 vs 10 ± 2.8), of which more were GV oocytes (7.2 ± 3.5 vs 5.1 ± 1.5, *P* = 0.009) (Table 3). Fertilization and blastulation outcomes per rMII oocytes were also significantly higher in this group of couples, in turn resulting in a larger number of 2PN zygotes (2.5 ± 1.1 vs 1.5 ± 1.3) and of blastocysts from rMII (1.2 ± 0.4 vs 0.03 ± 0.2). Conversely, the performance of the control MII oocytes was comparable in the two patients’ groups (Table 3).

**Table 3.** **Main differences between patients not obtaining or obtaining euploid blastocysts from rescued metaphase II (rMII) oocytes**.

	Patients not obtaining euploid from rMII (N = 34)	Patients obtaining euploid from rMII (N = 13)	*P*-value
Age (years) mean ± SD	35.9 ± 2.3	35.7 ± 2.7	0.942
AMH (ng/ml) mean ± SD	2.1 ± 1.8	2.4 ± 1.3	0.397
AFC mean ± SD	11.2 ± 4.7	12.2 ± 4.7	0.251
Main cause of infertility, n/N, %			*P* = 0.854
Idiopathic	5/34, 14.7%	3/13, 23.1%	
AMA	23/34, 67.6%	9/13, 69.2%	
Endometriosis	2/34, 5.9%	0/13, 0%	
Adenomyosis	1/34, 2.9%	0/13, 0%	
Tubal	1/34, 2.9%	0/13, 0%	
Reduced ovarian reserve	2/34, 5.9%	1/13, 7.7%	
Severe male factor, n/N, %			*P* = 0.569
No	27/34, 79.4%	10/13, 76.9%	
Yes	7/34, 20.6%	3/13, 23.1%	
Ovulation trigger, n/N, %			*P* = 0.876
hCG	9/34, 26.5%	3/13, 23.1%	
GnRH-a	17/34, 50%	6/13, 46.2%	
Double trigger	8/34, 23.5%	4/13, 30.8%	
Follicles ≥14 mm at ovulation trigger mean ± SD	8.4 ± 2.3	10.6 ± 5.8	0.157
FORT (follicles ≥14 mm at ovulation trigger/AFC) mean ± SD	82.2% ± 24.5%	87% ± 19.9%	0.534
COCs			**0.008**
N	340	176	
mean ± SD	10 ± 2.8	13.5 ± 5.1	
FOI (COCs/AFC) mean ± SD	121.4% ± 23.4%	139.9% ± 40.9%	0.058
Sibling control MII retrieved			0.070
N mean ± SD	140 4.1 ± 1.8	66 5.1 ± 1.1	
Recovery rate (sibling control MII/follicles ≥14 mm at ovulation trigger)			0.280
Overall	140/286, 48.9%	66/138, 47.8%	
Mean ± SD	47.7% ± 18.4%	53.9% ± 14.6%	
GV retrieved			**0.009**
N	172	94	
Mean ± SD	5.1 ± 1.5	7.2 ± 3.5	
GV rate			0.591
Overall	172/340, 50.6%	94/176, 53.4%	
Mean ± SD	52.2% ± 13.5%	52.8% ± 12.0%	
rMII			0.141
N	85	42	
Mean ± SD	2.5 ± 1.5	3.2 ± 1.3	
Rescue rate			0.971
Overall	85/172, 49.4%	42/94, 44.7%	
Mean ± SD	54.0% ± 34.5%	52.9% ± 26.8%	
Total MII (sibling control MII + rMII)			**0.031**
N	225	108	
Mean ± SD	6.6 ± 2.5	8.3 ± 1.8	
2PN from sibling control MII			
N	103	44	0.303
Mean ± SD	3.0 ± 1.4	3.4 ± 1.6	
2PN from rMII			**0.012**
N	51	33	
Mean ± SD	1.5 ± 1.3	2.5 ± 1.1	
Total 2PN			**0.014**
N	154	77	
Mean ± SD	4.5 ± 1.9	5.9 ± 1.9	
Sibling control MII fertilization rate			0.374
Overall	103/140, 73.6%	44/66, 66.7%	
Mean ± SD	77% ± 22.7%	64.9% ±31.2%	
rMII fertilization rate			**0.037**
Overall	51/85, 60%	33/42, 78.6%	
Mean ± SD	57.4% ±36.1%	82.4% ±19.3%	
Blastocysts from sibling control MII			0.384
N	49	23	
Mean ± SD	1.4 ± 1.2	1.8 ± 1.3	
Blastocysts from rMII			
N	1	16	**<0.001**
Mean ± SD	0.03 ± 0.2	1.2 ± 0.4	
Total blastocysts			**0.002**
N	50	39	
Mean ± SD	1.5 ± 1.3	3 ± 1.4	
Sibling control MII blastulation rate			0.574
Overall	49/103, 47.6%	23/44, 52.3%	
Mean ± SD	45.5% ± 33.1%	52.0% ± 28.5%	
rMII blastulation rate			**<0.001**
Overall	1/51, 1.96%	16/33, 48.5%	
Mean ± SD	2.1% ± 10.2%	54.5 ± 22.8%	

AMH, anti-Müllerian hormone; AFC, antral follicle count; AMA, advanced maternal age; GnRH-a, gonadotropin-releasing hormone agonist; FORT, follicle output ratio; FOI, follicle to oocyte index; COCs, cumulus oocyte complexes; GV, germinal vesicle; 2PN, two pronuclei; MII, Metaphase II; rMII, rescued metaphase II oocytes. Statistically significant *P*-values are reported in with a bold font.

Supplementary Figure S2A–D outlines the association between the number of MII oocytes and GV oocytes obtained among the PGT cycles clustered according to whether a euploid blastocyst was obtained or not. A logistic regression analysis showed that each additional GV oocyte submitted to the rescue protocol results in a 49% relative increase in the chance to obtain a euploid blastocyst from this practice (95% CI 1.05–2.12, *P* = 0.026).

### Morphometric and morphodynamic profiles of rescued GV oocytes

**Figure 5.**
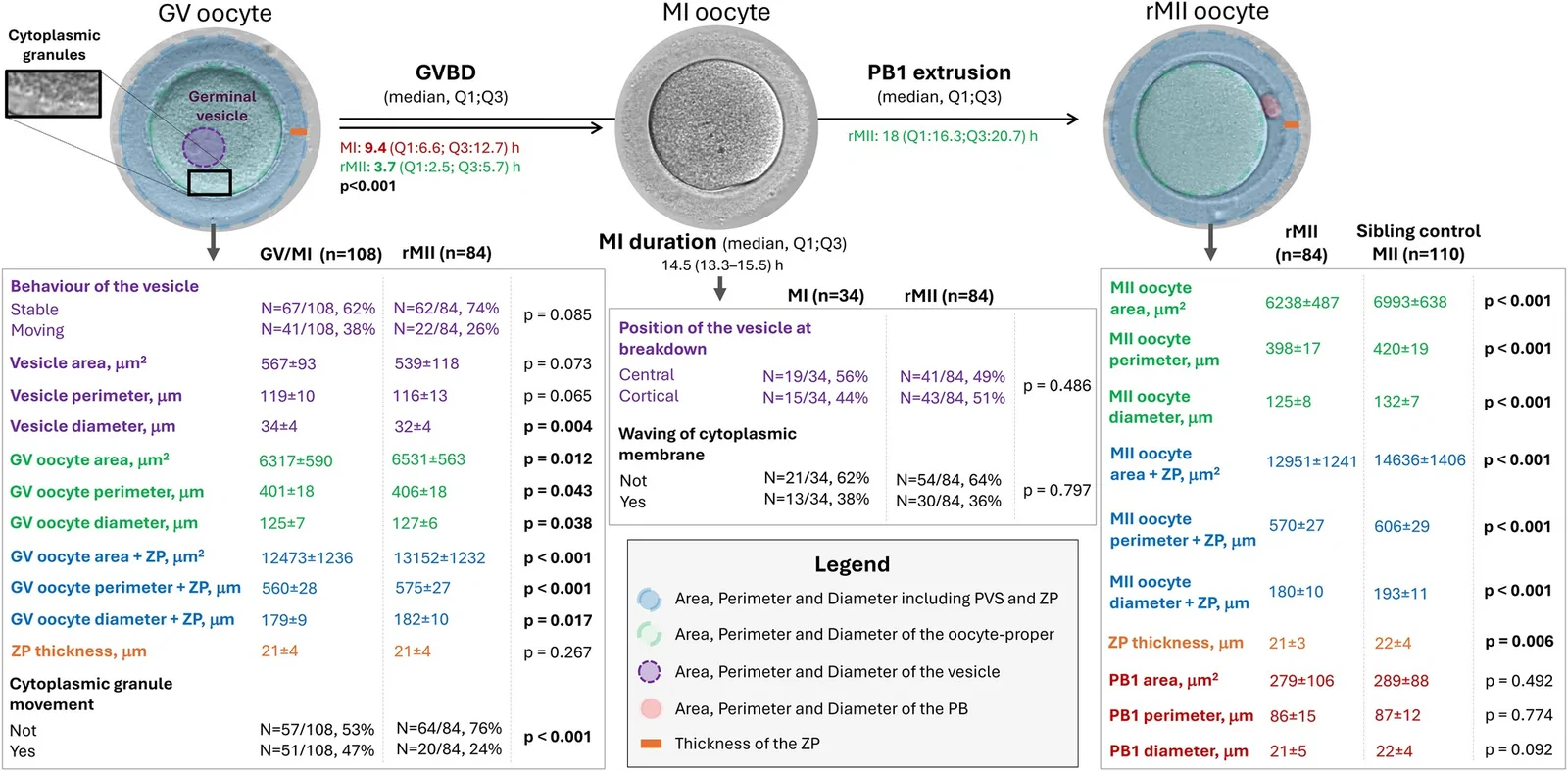
**Schematic overview of the morphometric and morphodynamic analyses performed during oocyte maturation**. Representative images of germinal vesicle (GV), metaphase I (MI), and rescue-metaphase II (rMII) oocytes are shown together with the timing of germinal vesicle breakdown (GVBD), MI duration, and first polar body (PB1) extrusion. Morphodynamic variables included vesicle behaviour (stable or moving), vesicle position at breakdown (central or cortical), cytoplasmic granule movement, and waving of the cytoplasmic membrane (examples shown in Supplementary Fig. S1A–C). Morphometric measurements included area, perimeter, and diameter of the vesicle, oocyte, oocyte including zona pellucida (ZP) and perivitelline space (PVS), and PB1. ZP thickness was also annotated. Data are reported as median with first and third quartiles (Q1–Q3; GVBD and PB1 extrusion times) or mean ± SD, together with sample sizes and *P*-value for relevant.

A total of 192 GV videos were included in this sub-analysis. Of these, 108 GV oocytes (56%) either remained arrested at the GV stage (n = 74) or progressed only to MI (n = 34), whereas 84 oocytes (44%) successfully completed *in vitro* maturation to the rMII stage. Morphodynamic and morphometric outcomes were analysed according to the two comparisons illustrated in Fig. 5. In the left panel of Fig. 5, GV oocytes that arrested prior to MII or reached at most MI were compared with rMII oocytes. No significant differences were observed in vesicle behaviour between groups, with a comparable distribution of stable and moving vesicles in GV/MI versus rMII. Similarly, vesicle positioning at breakdown did not differ significantly, with a similar proportion of central and cortical localization. Morphometric analysis showed comparable vesicle area and perimeter, while GV diameter was slightly but significantly larger in GV/MI oocytes (34 ± 4 µm vs 32 ± 4 µm; *P* = 0.004). In contrast, successfully rescued GV oocytes exhibited larger oocyte dimensions compared with arrested/MI counterparts, including greater area (6317 ± 590 µm^2^ vs 6531 ± 563 µm^2^; *P* = 0.012), perimeter (401 ± 18 µm vs 406 ± 18 µm; *P* = 0.043), and diameter (125 ± 7 µm vs 127 ± 6 µm; *P* = 0.038). When oocyte and ZP measurements were considered together, all parameters remained significantly lower in the GV/MI group: area (12 473 ± 1236 µm^2^ vs 13 152 ± 1232 µm^2^; *P* < 0.001), perimeter (560 ± 28 µm vs 575 ± 27 µm; *P* < 0.001), and diameter (179 ± 9 µm vs 182 ± 10 µm; *P* = 0.017). ZP thickness was comparable between groups. rMII oocytes also showed a lower prevalence of cytoplasmic granularity compared with GV/MI oocytes (n = 20/84, 24% vs n = 51/108, 47%; *P* < 0.001). Finally, when comparing GV oocytes that resumed meiosis but arrested at MI (n = 34) with those successfully rescued to MII, the latter showed significantly faster GVBD (3.7 h [Q1: 2.5; Q3: 5.7] vs 9.7 h [Q1: 6.6; Q3: 12.7]; *P* < 0.001). In successfully matured oocytes, the duration of MI was 14.5 h (Q1: 13.3; Q3: 15.5), while the time to PB1 extrusion was 18 h (Q1: 16.3; Q3: 20.7). Regarding membrane distortion during PB1 extrusion, it was observed in 15% (n = 13/84) of oocytes in the rMII group, while it was absent in 85% (n = 71/84). No significant differences were observed in oocytes showing waving cytoplasmic membrane between MI-arrested oocytes and rMII. In the right panel of Fig. 5, morphometric analysis showed that rMII oocytes exhibited significantly lower values compared with sibling controls MII for all oocyte-related parameters, including area (6238 ± 487 µm^2^ vs 6993 ± 638 µm^2^; *P* < 0.001), perimeter (398 ± 17 µm vs 420 ± 19 µm; *P* < 0.001), and diameter (125 ± 8 µm vs 132 ± 7 µm; *P* < 0.001). Similar differences were observed when considering the oocyte plus ZP, with reduced area (12 951 ± 1241 µm^2^ vs 14 636 ± 1406 µm^2^; *P* < 0.001), perimeter (570 ± 27 µm vs 606 ± 29 µm; *P* < 0.001), and diameter (180 ± 10 µm vs 193 ± 11 µm; *P* < 0.001) in rMII oocytes. ZP thickness was also slightly lower in rMII compared with controls (21 ± 3 µm vs 22 ± 4 µm; *P* = 0.006). In contrast, PB1 morphometry did not differ significantly between groups. A multivariate logistic regression analysis was conducted to confirm the association between morphodynamic and morphometric parameters and oocyte maturation following GV rescue (Table 4). Delayed GVBD was significantly associated with lower odds of rescue (OR = 0.719, 95% CI: 0.542–0.955; *P* = 0.023), while larger GV oocytes’ area including the ZP was significantly associated with higher odds (OR = 1.078, 95% CI: 1.021–1.139 every 100 µm^2^-increase; *P* = 0.007).

**Table 4.** **Multivariate logistic regression analysis of factors associated with the oocyte maturation outcome following germinal vesicle (GV) rescue**.

	Multivariate-OR, 95% CI, *P*-value
Cytoplasmic granule movement	0.942, 95% CI 0.214–4.156, *P* = 0.938
Timing GVBD	**0.719, 95% CI 0.542–0.955, *P* = 0.023**
GV oocyte area + ZP (per 100 µm² increase)	**1.078, 95% CI 1.021–1.139, *P* = 0.007**

OR, odds ratio; GVBD, germinal vesicle breakdown; ZP, zona pellucida. Statistically significant associations are reported with a bold font.

rMII oocytes were stratified according to developmental outcome as developmentally competent (BL+, oocytes reaching the blastocyst stage) and developmentally incompetent (BL−, oocytes failing to reach blastocyst). Analyses were performed for rMII BL+ versus rMII BL−, and rMII BL+ versus sibling BL+ controls (Supplementary Fig. S3). Within the rMII cohort, no significant differences were observed in oocyte morphometric parameters between BL+ and BL− oocytes, including MII oocyte area, perimeter, and diameter. Oocyte plus ZP measurements were also comparable, as it was ZP thickness. No difference was observed in PB1 morphometrics between BL+ and BL− oocytes and membrane distortion during its extrusion showed no apparent association.

In the comparison between rMII BL+ and sibling control BL+ MII oocytes, the latter exhibited larger MII oocyte area (6941 ± 546 vs 6346 ± 280 µm^2^; *P* = 0.006), perimeter (418 ± 17 vs 399 ± 9 µm; *P* = 0.007), and diameter (131 ± 6 vs 128 ± 5 µm; *P* = 0.017). ZP–inclusive area was also significantly larger (14 713 ± 1355 vs 13 306 ± 1593 µm^2^; *P* = 0.011), whereas ZP thickness was similar between groups. PB1 perimeter differed modestly but significantly (86 ± 12 vs 82 ± 7 µm; *P* = 0.020), while PB1 area and diameter were comparable.

Regarding developmental kinetics, PB2 extrusion timing was similar across groups. No significant difference was observed in PN appearance or fading between rMII BL+ and sibling control BL+ MII oocytes. Conversely, rMII BL+ oocytes exhibited earlier PN juxtaposition [(8.6 h (Q1: 7.2; Q3: 8.9) vs 11.2 h (Q1: 8; Q3: 12.7); *P* = 0.046)] and reduced time to cleavage [(24.8 h (Q1: 22.9; Q3: 27.1) vs 28.1 h (Q1: 25.7; Q3: 32.2); *P* = 0.046)] compared with rMII BL− oocytes; PN dynamics or cleavage timing were instead comparable. Compared with sibling control MII oocytes, blastocyst formation was markedly reduced in the rMII group (N = 6). Most embryos derived from rMII oocytes were either unfertilized or underwent early degeneration following fertilization (N = 24). Among the remaining embryos, 11 arrested at developmental stages ≤8 cells, whereas only 7 progressed beyond the 8-cell stage, precluding reliable downstream analyses.

## Discussion

This study reports the 8-year clinical experience with GV rescue in a highly selected population of poor prognosis patients showing an unexpectedly low (∼50%) mature oocytes yield despite normal baseline characteristics (age, AMH, AFC, and ≥14 mm follicles at trigger), a rate largely lower than the 80% benchmark set by ESHRE (ESHRE and Alpha, 2017) and in line with a data-driven warning threshold outlined in a recent study (Taggi *et al.*, 2026). Building on a previous study from our group (Escrich *et al.*, 2018), which demonstrated the feasibility and potential benefits of GV rescue, this observational study adopted strict inclusion criteria and high methodological consistency throughout the study period. If on the one hand, this choice largely limited the sample size, on the other hand, it ensured the cautious selection of a population of patients who might truly benefit from a still controversial procedure, applying it in a setting that also involved comprehensive chromosome testing. As a result, only 47 women were included, which represent 1.7% of all PGT-A cycles conducted during the study period at our centre, in line with previous reports (Ben-Shlomo *et al.*, 1991; Işik and Vicdan, 2000; Zreik *et al.*, 2000; Nikolettos *et al.*, 2004; Aktas *et al.*, 2005; Duru *et al.*, 2007; Desai *et al.*, 2009; Vutyavanich *et al.*, 2010; Castillo *et al.*, 2020). An unexpected suboptimal maturation rate is typically faced by inseminating only the MII oocytes or vitrifying them and running a second ovarian stimulation in the short term. GV rescue might represent a third valuable option. In this study, we reported a positive contribution of GV rescue to larger cohorts of oocytes, zygotes, blastocysts, and euploid blastocysts, and a relative increase in the chance of obtaining blastocysts of 8%, euploid blastocysts of 15%, and a live birth of 20%. This contribution was achieved with a limited additional workload for the laboratory, namely 42 additional ICSI procedures, 17 additional biopsies and vitrifications, and 10 additional warmings and embryo transfers. A more thorough analysis of the additional workload and of the cost-effectiveness of GV rescue is certainly warranted, but these data are promising.

From a comparison between the 13 patients who obtained euploid blastocysts from GV rescue versus the 34 who did not, the former were characterized by higher ovarian sensitivity indexes and collected more COCs, obtaining on average a larger number of GV oocytes with respect to the latter. No difference was reported in terms of sibling control MII oocytes’ developmental competence, while the GV cohorts showed better outcomes. Overall, the logistic regression analysis outlined a 49%-relative increase in the chance to obtain ≥1 euploid blastocyst for each additional GV oocyte submitted to the rescue protocol, suggesting that this protocol might be particularly advantageous for patients characterized by a larger number and proportion of immature oocytes after ovarian stimulation.

The extent of benefit from GV rescue is largely dependent on its main limiting factor, namely the rescue rate itself. Despite the lack of a standardized GV rescue protocol, several studies have explored optimization of culture media, supplements, and maturation duration to improve both nuclear and cytoplasmic competence (Soler *et al.*, 2025). Building on our previous experience and using time-lapse technology, we selected rMII oocytes that completed meiosis within 24 h of culture. The observed rescue rate (∼57%) and fertilization rate (∼66%) were in line with previous findings reported using artificial oocyte activation (Soler *et al.*, 2025), and the latter outcome was not significantly different from sibling control MII oocytes. Blastulation rates were instead considerably lower, as consistently shown also by previous reports (Bartolacci *et al.*, 2024). Nevertheless, the resulting blastocysts showed comparable morphology and chromosomal constitution than control embryos. Indeed, oocyte competence comprehensively assessed as euploid blastocyst rate per inseminated oocyte did not result in statistically significant differences, further supporting the clinical use of GV rescue in very poor prognosis patients. In a recent larger study from another group (Elkhatib *et al.*, 2023), rIVM resulted in euploidy rates at the blastocyst stage similar to those in sibling control embryos, thereby contributing meaningfully to the available blastocyst cohort, particularly in poor-prognosis patients characterized by low oocyte yield and reduced maturation rates. Based on these data, PGT-A is not necessarily indicated in the context of rIVM and its adoption should follow the same indications as for standard IVF cycles. Although limited, the clinical outcomes per euploid embryo transfer, including gestational age and birthweight, obtained in our study are promising and encourage further investigations on this rescue strategy in broader population of patients and adopting less restrictive inclusion criteria. To this end, a recent paper suggested that an immaturity rate ≥50% with ≥5 COCs is a statistically sound threshold to candidate a couple to this practice, especially if occurring across multiple ovarian stimulations (Taggi *et al.*, 2026). This is a criterion worth exploring by applying rIVM clinically.

Further insight into the determinants of oocyte competence within the GV rescue setting was provided by morphodynamic and morphometric analysis of GV oocytes. Cytoplasmic granule movement, although not an independent predictor in the multivariate analysis, was more frequently observed in oocytes that failed to complete maturation compared with successfully rescued GV oocytes. This finding is consistent with ultrastructural studies describing cytoplasmic granularity as a manifestation of altered subcellular organization, including mitochondrial redistribution and endoplasmic reticulum clustering, typically associated with incomplete or asynchronous cytoplasmic maturation (Palmerini *et al.*, 2022). Importantly, abnormalities of oocyte maturation have been reported to reflect a dissociation between nuclear and cytoplasmic maturation processes, whereby meiotic progression may occur despite suboptimal cytoplasmic organization, which may explain the lack of independent predictive value of cytoplasmic dysmorphisms (Baldini *et al.*, 2024). A shorter GVBD interval was significantly associated with successful maturation in our GV rescue cohort, suggesting that the kinetics of meiotic resumption reflect intrinsic oocyte competence. This observation is consistent with previous clinical evidence showing that GVBD dynamics are associated with reproductive outcomes in ICSI cycles with surplus immature oocytes, supporting its role as a marker of developmental potential (Escrich *et al.*, 2012; Wang *et al.*, 2022; Soler *et al.*, 2025). Mechanistically, GVBD reflects the integration of oocyte-intrinsic signalling and cumulus–oocyte communication pathways, including metabolic regulation and oxidative stress responses mediated in part by AMPK signalling (Ban *et al.*, 2025). Accordingly, faster GVBD likely reflects more efficient cytoplasmic maturation and a more favourable metabolic state, facilitating progression to rMII. Finally, GV oocytes reaching the rMII stage were larger than those arresting at the GV/MI stage, whereas the rMII oocytes they produced remained smaller than sibling control MII oocytes. Studies in large animals indicated that morphometry alone is insufficient to fully capture oocyte competence whereas, if integrated with morphometric and gene expression profiling of oocytes and cumulus cells, it might provide a more robust indicator of developmental potential (Maside *et al.*, 2021). In this regard, our investigation of fertilization morphodynamics after rMII insemination showed a positive association between earlier PN juxtaposition and cleavage with blastocyst development, in line with previous evidence in animal models and humans (Cavazza *et al.*, 2021; Coticchio *et al.*, 2023). Overall, these preliminary morphometric and morphodynamic observations encourage future validation in larger and adequately powered cohorts. In this context, artificial intelligence-powered pipelines can support the automated and faster acquisition of reliable data to train future predictive tools.

### Limitations and future perspectives

This single centre observational study is limited by a low sample size. Nevertheless, the evidence generated in this dataset of 266 GV oocytes from 47 patients is highly promising and paves the way to future investigations on a controversial clinical strategy which is again under the spotlight for its potential benefits in highly selected populations of poor prognosis patients. Four live births were achieved from 10 vitrified-warmed euploid blastocyst transfers. More evidence is certainly needed to highlight neonatal outcomes and long-term follow-up.

An additional limitation of this study is that rescue maturation excluded MI oocytes and restricted the GV rescue to a 24-h threshold. These choices were driven by previous clinical experience that informed this study protocol design (Escrich *et al.*, 2012). Specifically, from a lab management perspective, in metaphase-I oocytes the exact timing of PB1 extrusion is highly heterogeneous and unpredictable, as it may occur in the evening or night, outside of the standard working hours. Therefore, ICSI would most likely be postponed to the following morning, introducing a delay that may induce post-ovulatory *in vitro* aging and further compromise oocyte developmental competence, thereby representing a relevant confounder for outcome assessment. Conversely, GV oocytes require 18 h (Q1: 16, Q3: 21) to progress to the rMII stage, making their rescue a reasonable compromise between lab workload management and putative clinical benefit.

In the future, alternative rIVM strategies preserving oocyte–cumulus cell communication might be envisioned. In fact, increasing evidence indicates that cumulus cells actively contribute to oocyte competence through paracrine signalling, including the release of extracellular vesicles containing miRNAs that regulate key pathways involved in the acquisition of oocyte developmental competence during the GV-to-MII transition in mouse (Fiorentino *et al.*, 2024). A mechanism that seems conserved also in humans (Brazzale *et al.*, 2025). This scenario envisions a future where specific molecules might be supplemented to the rescue medium to improve oocyte developmental competence, as it has been preliminarily shown with nicotinamide mononucleotide (NMN) in mouse and human oocytes (Ramírez-Martín *et al.*, 2025).

## Conclusions

GV rescue represents a patient-centred strategy that can increase cumulative IVF success in cycles with limited mature oocyte yield. Further refining of patient selection criteria and standardizing this approach may enhance its clinical utility in future appraisals.

## Supplementary Material

deag115_Supplementary_Figure_S1

deag115_Supplementary_Figure_S2

deag115_Supplementary_Figure_S3

## Data Availability

The data underlying this article are available in the article and in its online supplementary material.
