# Supplementary material for: Germinal vesicle oocytes’ rescue: a worthless treatment or a patient-centred approach? Eight years of clinical experience in poor prognosis patients
Source: Hum Reprod. 2026 Jul 13;41(9):1507–21. doi: 10.1093/humrep/deag115 (PMC13550666; doi:10.1093/humrep/deag115)
Supplement: Supplementary file 1 [file deag115_supplementary_figure_s1.pdf]

Human Reproduction, 2026, 1–1
https://doi.org/10.1093/humrep/deag115
Supplementary Data

human reproduction OXFORD

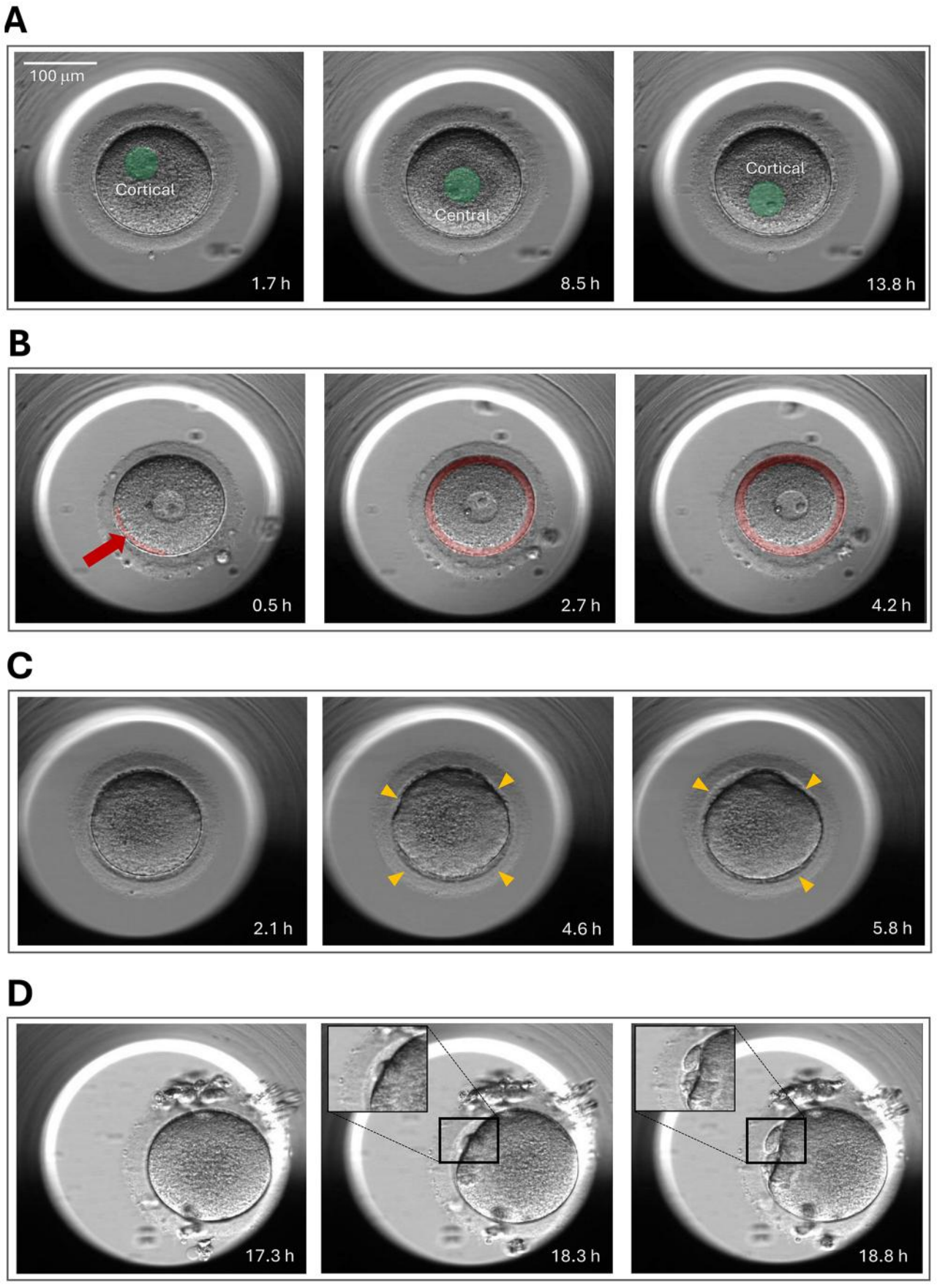

Supplementary Figure S1. Morphodynamic behaviours during germinal vesicle (GV) oocytes' rescue. (A) Representative time-lapse sequence illustrating the behaviour of a moving GV (green) prior to germinal vesicle breakdown (GVBD). The specific position as central or cortical is highlighted. **(B)** Representative time-lapse sequence illustrating cytoplasmic granule movement before GVBD. Red halos outline the oocytes areas without cytoplasmic granules. **(C)** Representative time-lapse sequence illustrating the waving of the cytoplasmic membrane during the MI (metaphase I) stage (yellow arrowheads). **(D)** Representative time-lapse sequence illustrating membrane distortion during first polar body (PB1) extrusion (inserts). Scale bar = 100 µm.
