# Supplementary material for: Germinal vesicle oocytes’ rescue: a worthless treatment or a patient-centred approach? Eight years of clinical experience in poor prognosis patients
Source: Hum Reprod. 2026 Jul 13;41(9):1507–21. doi: 10.1093/humrep/deag115 (PMC13550666; doi:10.1093/humrep/deag115)
Supplement: Supplementary file 2 [file deag115_supplementary_figure_s2.pdf]

Human Reproduction, 2026, 1–1
https://doi.org/10.1093/humrep/deag115
Supplementary Data

human reproduction
OXFORD

A

Cycles without euploid from rMII oocytes		Number of GV oocytes 4	5	6	7	8	10	14	15	TOT cycles
Number of MII oocytes	0	1	0	0	0	0	0	0	0	*1*
	1	1	2	0	0	0	0	0	0	*3*
	2	3	0	0	1	0	0	0	0	*4*
	3	1	0	0	0	0	0	0	0	*1*
	4	5	0	1	1	0	0	0	0	*7*
	5	5	3	1	1	0	0	0	0	*10*
	6	3	0	1	2	1	1	0	0	*8*
TOT cycles		*19*	*5*	*3*	*5*	*1*	*1*	*0*	*0*	*34*

B

Cycles with euploid only from rMII oocytes		Number of GV oocytes 4	5	6	7	8	10	14	15	TOT cycles
Number of MII oocytes	0	0	0	0	0	0	0	0	0	*0*
	1	0	0	0	0	0	0	0	0	*0*
	2	0	1	0	0	0	0	0	0	*1*
	3	0	0	0	0	0	0	0	0	*0*
	4	0	0	0	0	0	0	0	0	*0*
	5	1	0	0	0	0	0	1	0	*2*
	6	1	0	0	0	0	0	0	0	*1*
TOT cycles		*2*	*1*	*0*	*0*	*0*	*0*	*1*	*0*	*4*

C

Cycles with euploid also from rMII oocytes		Number of GV oocytes 4	5	6	7	8	10	14	15	TOT cycles
Number of MII oocytes	0	0	0	0	0	0	0	0	0	*0*
	1	0	0	0	0	0	0	0	0	*0*
	2	0	0	0	0	0	0	0	0	*0*
	3	0	0	0	0	0	0	0	0	*0*
	4	0	0	0	0	1	0	0	0	*1*
	5	0	1	1	1	1	0	0	0	*4*
	6	0	2	0	0	1	0	0	1	*4*
TOT cycles		*0*	*3*	*1*	*1*	*3*	*0*	*0*	*1*	*9*

D

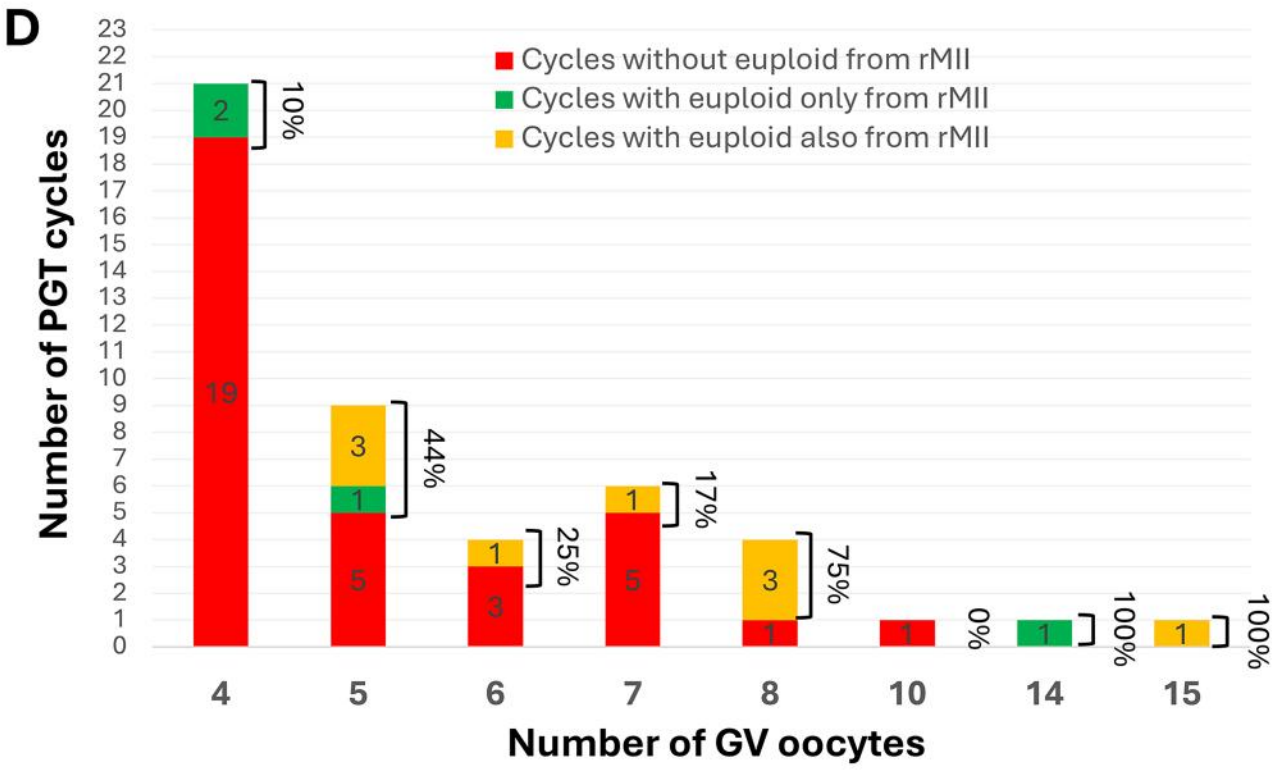

Increase in the GV oocytes undergoing rescue vs chance to obtain an euploid blastocyst: OR 1.49, 95%CI 1.05-2.12, p=0.026

Supplementary Figure S2. Correlation matrix showing the association between the number of metaphase II oocytes (MII) and of germinal vesicle (GV) oocytes among preimplantation genetic testing (PGT) cycles where (A) no euploid blastocyst was obtained from GV rescue (N = 34) (B) euploid blastocysts were obtained only from rMII (MII derived from GV rescue; N = 4), (C) euploid blastocysts were obtained also from rMII (N = 9). Figure (D) is a summary of the data in figure (A–C) accounting only for the number of GV oocytes obtained. The logistic regression analysis outlined that each additional GV oocyte submitted to a rescue procedure involved a 49% relative increase in the chance to obtain an euploid blastocysts.
