# Supplementary material for: Germinal vesicle oocytes’ rescue: a worthless treatment or a patient-centred approach? Eight years of clinical experience in poor prognosis patients
Source: Hum Reprod. 2026 Jul 13;41(9):1507–21. doi: 10.1093/humrep/deag115 (PMC13550666; doi:10.1093/humrep/deag115)
Supplement: Supplementary file 3 [file deag115_supplementary_figure_s3.pdf]

Human Reproduction, 2026, 1–1
https://doi.org/10.1093/humrep/deag115
Supplementary Data

human reproduction

OXFORD

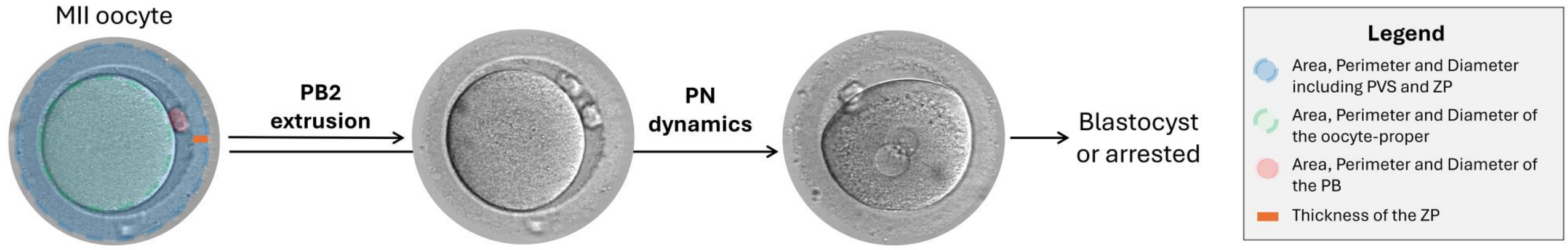

	rMII BL- (n=42)	rMII BL+ (n=6)	Sibling control MII BL- (n=68)	Sibling control MII BL+ (n=42)	*rMII BL– vs BL+*	*rMII BL+ vs Sibling control MII BL+*
**MII oocyte area, µm²**	6228±501	6346±280	7025±691	6941±546	p = 0.868	**p = 0.006**
**MII oocyte perimeter, µm**	398±18	399±9	421±20	418±17	p = 0.542	**p = 0.007**
**MII oocyte diameter, µm**	125±8	128±5	133±8	131±6	p = 0.432	**p = 0.017**
**MII oocyte area + ZP, µm²**	12919±1212	13306±1593	14588±1444	14713±1355	p = 0.469	**p = 0.011**
**MII oocyte perimeter + ZP, µm**	570±27	578±33	605±30	608±28	p = 0.919	p = 0.431
**MII oocyte diameter + ZP, µm**	180±10	184±14	193±11	195±11	p = 0.608	p = 0.355
**ZP thickness, µm**	21±3	21±1	22±4	22±4	p = 0.469	p = 0.374
**PB1 area, µm²**	281±109	259±55	287±87	293±91	p = 0.355	p = 0.129
**PB1 perimeter, µm**	86±15	82±7	87±13	86±12	p = 0.360	**p = 0.020**
**PB1 diameter, µm**	21±5	21±3	22±5	22±4	p = 0.852	p = 0.345
**PB2 extrusion, hours**	3.3 (Q1:2; Q3:4.1)	3.5 (Q1:2.4; Q3:4.1)	3.6 (Q1:3; Q3:4.3)	3.2 (Q1:2.4; Q3:3.8)	p = 0.813	p = 0.654
**Larger PNa, hours**	7.9 (Q1:2.9; Q3:4.9)	6.1 (Q1:5.1; Q3:6.8)	8.6 (Q1:7.5; Q3:10.5)	6.8 (Q1:6; Q3:7.5)	p = 0.127	p = 0.334
**Smaller PNa, hours**	7.9 (Q1:2.9; Q3:4.9)	6.1 (Q1:5.1; Q3:7.1)	8.4 (Q1:7.5; Q3:9.9)	6.7 (Q1:6.1; Q3:7.8)	p = 0.204	p = 0.474
**PN juxtaposition, hours**	11.2 (Q1:8; Q3:12.7)	8.6 (Q1:7.2; Q3:8.9)	10.8 (Q1:9.3; Q3:12.5)	8.8 (Q1:7.8; Q3:11.4)	**p = 0.046**	p = 0.188
**Larger PNf, hours**	23.7 (Q1:21.4; Q3:28)	22.2 (Q1:20.6; Q3:24.1)	25.1 (Q1:22.5; Q3:29.5)	23.3 (Q1:21.8; Q3:25.6)	p = 0.204	p = 0.280
**Smaller PNf, hours**	23.8 (Q1:21.4; Q3:28)	22.2 (Q1:20.6; Q3:24.1)	25.3 (Q1:23; Q3:30)	23.3 (Q1:21.8; Q3:25.6)	p = 0.162	p = 0.269
**Cleavage, hours**	28.1 (Q1:25.7; Q3:32.2)	24.8 (Q1:22.9; Q3:27.1)	27.7 (Q1:26.2; Q3:31.8)	26.1 (Q1:24.5; Q3:28.2)	**p = 0.046**	p = 0.344

Supplementary Figure S3. Morphometric and morphodynamic characterization of rescue-metaphase II (rMII) oocytes and sibling control MII oocytes according to developmental outcome. Representative images illustrate the sequential developmental events analysed following fertilization, including second polar body extrusion (PB2 extrusion), and pronuclei (PN) dynamics, leading either to blastocyst formation (BL+) or developmental arrest (BL–). Morphometric measurements included area, perimeter, and diameter of the oocyte, oocyte including zona pellucida (ZP), and PB1. ZP thickness was also annotated. Morphodynamic analyses included the timing of PB2 extrusion, larger and smaller pronuclear appearance (PNa), pronuclear juxtaposition, larger and smaller pronuclear fading (PNf), and cleavage. Data are presented as mean ± SD or median with first and third quartiles (Q1–Q3), together with sample sizes and *P*-value for relevant comparisons.
